# Automating analysis of vegetation with computer vision: Cover estimates and classification

**DOI:** 10.1002/ece3.4135

**Published:** 2018-05-15

**Authors:** Chris McCool, James Beattie, Michael Milford, Jonathan D. Bakker, Joslin L. Moore, Jennifer Firn

**Affiliations:** ^1^ School of Electrical Engineering and Computer Science Queensland University of Technolgy (QUT) Brisbane Qld Australia; ^2^ School of Environmental and Forest Sciences University of Washington Seattle Washington; ^3^ School of Biological Sciences Monash University Clayton Vic. Australia; ^4^ School of Earth Environmental and Biological Sciences, Queensland University of Technolgy (QUT) Brisbane Qld Australia

**Keywords:** automation, computer vision, image analysis, visual cover estimate

## Abstract

This study develops an approach to automating the process of vegetation cover estimates using computer vision and pattern recognition algorithms. Visual cover estimation is a key tool for many ecological studies, yet quadrat‐based analyses are known to suffer from issues of consistency between people as well as across sites (spatially) and time (temporally). Previous efforts to estimate cover from photograps require considerable manual work. We demonstrate that an automated system can be used to estimate vegetation cover and the type of vegetation cover present using top–down photographs of 1 m by 1 m quadrats. Vegetation cover is estimated by modelling the distribution of color using a multivariate Gaussian. The type of vegetation cover is then classified, using illumination robust local binary pattern features, into two broad groups: *graminoids* (*grasses*) and *forbs*. This system is evaluated on two datasets from the globally distributed experiment, the Nutrient Network (NutNet). These NutNet sites were selected for analyses because repeat photographs were taken over time and these sites are representative of very different grassland ecosystems—a low stature subalpine grassland in an alpine region of Australia and a higher stature and more productive lowland grassland in the Pacific Northwest of the USA. We find that estimates of treatment effects on *grass* and *forb* cover did not differ between field and automated estimates for eight of nine experimental treatments. Conclusions about total vegetation cover did not correspond quite as strongly, particularly at the more productive site. A limitation with this automated system is that the total vegetation cover is given as a percentage of pixels considered to contain vegetation, but ecologists can distinguish species with overlapping coverage and thus can estimate total coverage to exceed 100%. Automated approaches such as this offer techniques for estimating vegetation cover that are repeatable, cheaper to use, and likely more reliable for quantifying changes in vegetation over the long‐term. These approaches would also enable ecologists to increase the spatial and temporal depth of their coverage estimates with methods that allow for vegetation sampling over large spatial scales quickly.

## 1 INTRODUCTION

Point quadrat analyses and visual cover estimation of vegetation are widely used, standard vegetation survey techniques that date back to the 1920s (Gleason, 1920). These survey techniques are expensive and can be time‐consuming because they require an expert ecologist or botanist to make decisions about the amount and type of vegetation present. For these reasons, vegetation surveys are often conducted sparsely in terms of geographical location as well as temporally. In the case of the management of natural spaces on either private or public land, vegetation surveys and regular monitoring can be constrained by the availability of expert ecologists to be in the field with a reasonable assessment taking up to half a day per plot; survey time based on a plot size of 40 m^2^ (Vittoz & Guisan, 2007).

With long‐term research and monitoring, different ecologists may be required to conduct vegetation surveys over time, which increases the probabilities of inconsistencies in the accuracy of cover estimates made by multiple experts at the same site (Bergstedt, Westerberg, & Milber, 2009; Vittoz & Guisan, 2007). Vittoz and Guisan (2007) found that one ecologist completing a survey was more reliable than a succession of different ecologists. These issues motivated us to explore automated methods that could permit vegetation surveys to be conducted more frequently (temporally) and potentially over larger areas (spatially) with similar and, certainly for long‐term monitoring, better consistency to an expert.

Algorithms that interpret the visual imagery are a key for automating plot‐based vegetation surveys. Considerable work on detecting the presence of vegetation from imagery, and on classifying vegetation/weeds, has been conducted in the field of robotics, automation, and computer vision. In robotics, vegetation detection has been dominated by the use of vegetative indices that usually consist of a ratio between pixel values of color and/or near‐infrared (NIR) imagery (Haug, Michaels, Biber, & Ostermann, 2014; Keranen, Aro, Tyystjarvi, & Nevalainen, 2003; Weis et al., 2008). A downside of these approaches is that they often require specialized hardware as joint color and near‐infrared imagery is necessary. Outside of vegetative indices, researchers have explored transforming the raw color information (RGB) to a more suitable color space. Philipp and Rath (2002) found that the Lab, Luv, and HSV color spaces were particularly effective to discriminate between vegetation and nonvegetation.

Automated classification of vegetation is a challenge because of large intraclass and small interclass variations. Intraclass variation includes differences among growth stages of the same vegetation (species of plant), differences among species in the same vegetation type, and differences in growth form (shape, size) based on environmental conditions. Interclass variation can be small because species that are visually similar may belong to different vegetation types. For example, distinguishing a forb with long linear leaves (e.g., *Plantago lanceolata*) from a grass is a significant challenge. Successful applications of vegetation classification may require restricting the classification to particular subsets of plants. For example, Gerhards and Oebel (2006) distinguished between three different broadleaf weed species but grouped all grasses together. A variant of local binary patterns (LBPs) has also been used to classify leaf images for 51 species (Herdiyeni & Santoni, 2012) achieving good accuracy of 72% for this challenging multiclass problem. Haug et al. (2014) and Hung, Xu, and Sukkarieh (2014) approached this as a two‐class classification problem, rather than multiclass classification. Haug et al. (2014) performed *crop* versus *weed* classification in an agricultural setting and achieved high accuracy using shape and pixel statistic features. Hung et al. (2014) learnt features to classify *weeds* versus *not weeds* to detect invasive weeds, using imagery collected by an unmanned aerial vehicle (UAV), they were able to achieve high accuracy (>90%) for two of three weed species. Recently, an approach to detect and classify vegetation was developed by Bawden et al. (2017) for automatic robotic weeding which achieved an accuracy of 96.0% and 95.9% for classifying *grass* and *forbs,* respectively. However, this approach was only applied to simple weed detection in a monoculture crop.

Limited efforts have been made to apply such techniques in an ecological setting. One of the few examples is the work of Kendal et al. (2013) who used color threshold techniques for estimating vegetation cover using photographs. Kendal et al. demonstrated that their approach was promising for estimating total cover; however, it was not able to differentiate vegetation types, especially between species. Another limitation was that they required a ColorChecker board to be in the image so that the color in the images could be standardized. A reliable and consistent automated approach for estimating vegetation cover from visual imagery could revolutionize how we assess vegetation changes over time.

In this study, we take the approach of Bawden et al. (2017) and apply it to automate quadrat‐based studies. This approach can make use of nonspecialized or consumer‐level cameras, potentially even mobile phone imagery which is a near ubiquitous technology. As they rely on collecting imagery, the time spent in the field will be much less than a field ecologist performing vegetation surveys. Furthermore, such an approach has the advantage of being deployable on many lightweight robotic platforms (including unmanned aerial vehicles), as well as being usable by citizen–scientists.

We apply the automated system to digital SLR imagery and evaluate its efficacy to: (1) detect vegetation, and (2) classify vegetation into two broad groups (classes), *grasses* and *forbs*. Overall, we find grass and forb cover estimates are correlated with estimates made in‐situ by field ecologists. We then consider the implications of these estimates for determining differences among experimental treatments; in many cases, the same conclusions were arrived at whether based on estimates from the automated system or from field ecologists.

## 2 AUTOMATIC ESTIMATION OF VEGETATION COVER

We present an automated approach^1^ to detect and classify vegetation using still camera images. The proposed algorithms are evaluated on quadrat images taken by a field ecologist, using a top–down view of 1 m by 1 m quadrats. These images are taken at the same time that the field ecologist estimates the amount and type of vegetation present. This provides us with a catalogue of images with associated ground truth estimates of the amount and type of vegetation recorded by the ecologist in the field.

An assumption made is that the images taken from a site are consistent. This means that a similar camera was used to take the images and that the scale and pose of the camera was similar; the images were taken at a similar distance with a similar angle.

The performance of our proposed algorithms is compared against the question that is central for this study: “Can images from consumer‐level cameras be analyzed to automatically estimate important coverage values”? To this end, we only evaluate the performance of our system against the result of the in‐field ecologist (ground truth) for three key tasks: (1) estimating total vegetation cover, (2) estimating the total cover of vegetation type (grasses vs. forbs), and (3) the effect that a particular treatment had on the environmental system. As we concentrate on this central question, we do not explore engineering approaches to incrementally improve performance such as data augmentation (Krizhevsky, Sutskever, & Hinton, 2012; Poh, Marcel, & Bengio, 2003). More details on this dataset can be found in Section 3. Below, we outline the automated approaches for detecting and then classifying vegetation.

### 2.1 Vegetation detection

To detect vegetation, we model the distribution of vegetation color using a multivariate Gaussian. We use a pretrained model as described in Bawden et al. (2017). The model is trained on data not taken from the quadrat images to ensure its generality for detecting (green) vegetation. This enables it to be deployed quickly and easily to different settings. The detection model was trained, evaluated, and tested using 40 images taken with two cameras: a Canon 7D and a mobile phone (Sony XPeria Z3 Compact). The 40 images were split such that 14 images were used for training, 14 for evaluation, and 12 for testing; The DSLR camera had an image resolution of 2,592 × 1,728 and the mobile phone had an image resolution of 3,840 × 2,160. Below we describe the approach taken, further details of this approach can also be found in Bawden et al. (2017).

The standard RGB representation of each pixel is first transformed to other well‐known color spaces. We make use of the Lab, Luv, and HSV color spaces as these are known to provide more consistent representations of color than standard RGB. Each of these new color spaces consists of two chromaticity components and an intensity component (either L or V). To provide robustness to varying illumination conditions, we remove the intensity components by ignoring them and combine the three representations into a *D* = 6 dimensional feature vector ***z***.

The color of green vegetation is then modelled using a multivariate Gaussian, and is standard irrespective to the photograph imaging equipment. This model combines the information from all *D* = 6 dimensions of the feature vector, ***z***, and is defined by its mean **μ** and covariance **Σ** (assumed to be diagonal as calculating the log‐likelihood of the diagonal function is the most efficient^2^). The likelihood that a pixel represents green vegetation is then given by,

$$p\left(z;\mu ,\sum \right)={\left(2\pi \right)}^{-\frac{1}{2}}\det{\left(\sum \right)}^{-\frac{1}{2}}\exp\left[-\frac{1}{2}{\left(z-\mu \right)}^{T}\sum^{-1}\left(z-\mu \right)\right].$$

A pixel is then declared as being green vegetation if its likelihood is greater than a predetermined threshold τ,

$$p\left(z;\mu ,\sum \right)=\left\{\begin{array}{cc} >\tau & \text{vegetation}\\ \leq \tau & \text{not vegetation} \end{array}.\right.$$

The parameters of this model, θ = [**μ**,** Σ**], are estimated on an independent training set of 14 annotated images,

$$\mu =\frac{1}{\mathrm{IXY}}\sum_{i=1}^{I}\sum_{x=1}^{X}\sum_{y=1}^{Y}z_{x,y}^{i}$$

$$\sum =\frac{1}{\mathrm{IXY}}\text{diag}\left(\sum_{i=1}^{I}\sum_{x=1}^{X}\sum_{y=1}^{Y}{\left(z_{x,y}^{i}-\mu \right)}^{T}\left(z_{x,y}^{i}-\mu \right)\right).$$

Where the operator diag(.) takes the diagonal of the resultant matrix. The predetermined threshold, τ, is calculated on an evaluation set of 14 annotated images and achieved an *F*_1_ score of 91.5 on the 12 annotated test image (Bawden et al., 2017). The *F*_1_ score is the point at which the precision, the ratio of selected pixels which are vegetation, and recall, the ratio of vegetation pixels which are correctly selected, are equal.

### 2.2 Vegetation classification

Once a pixel has been identified as containing vegetation, we classify the type of vegetation present using visual texture based on a region around the pixel. The visual texture feature is a histogram of LBPs which was shown by Bawden et al. (2017) to achieve an accuracy of 96.0% and 95.9% for classifying *grass* and *forbs*. The procedure in three steps.

1. For each pixel declared as being green vegetation, we take a two‐dimensional (2D) window around it and each pixel within the window is converted to an LBP.
2. We use a histogram of LBPs as the feature to compactly represent each 2D window. This histogram of LBPs is now our feature vector, ***y***_*k*_, which is used to represent the visual texture associated to the *k*‐th window, ***W***_*k*_.
3. The class for the *k*‐th window is compared against templates for each class (*grass* and *forb*) and the class template that best matches (represents) the window is declared as the vegetation type for that pixel.

**Figure 1:**
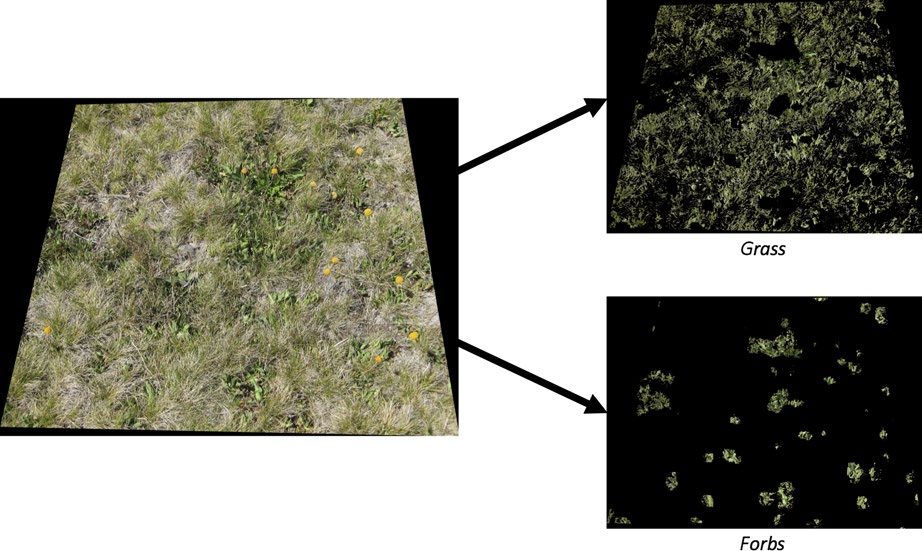
An example output of the vegetation classification procedure. On the left is the area within the quadrat and on the right, from top to bottom, is the result of grass and forb classification

An example of output of this procedure is given in Figure 1. Below, we describe each step in more detail.

For step 1, we use a square sliding window of *W *× *W* pixels, also known as a sliding window. This type of window is commonly used to detect faces and other objects. Here, we used *W *= 100 pixels. Each pixel within the window is converted to an LBP.

LBPs have been used for a range of computer vision tasks from visual texture classification through to face recognition (Ahonen, Hadid, & Pietikainen, 2006; Ojala, Pietikainen, & Maenpaa, 2002). They encode local texture information into a binary string consisting of *N* entries. These entries are obtained by comparing the central pixel *P*_c_ with *N* surrounding pixels sampled at a distance *R* from the central pixel. This leads to an illumination robust feature as local differences are used to obtain the feature.

The local binary pattern for pixel values can be interpreted as a binary string that can be converted to an integer value,

$${\text{LBP}}_{P,R}=\sum_{n=0}^{N-1}h\left(P_{n}-P_{c}\right)2^{n}$$

where *h*(*x*) is a binary function

$$h\left(x\right)=\left\{\begin{array}{ccc} 1 & & x\geq 0\\ 0 & & \text{otherwise.} \end{array}\right.$$

This process is applied to each pixel. If pixels are on the edge, then they are reflected but this process uses the imagery from the quadrat so edges are not an encountered issue.

For step 2, we use a histogram of LBPs to compactly represent each 2D window. This is achieved by summarizing the *W* × *W* LBP values from each 2D window as a histogram. The total number of histogram bins is *N* = 256 for this work. Thus, the *k*‐th window, ***W***_*k*_, is represented by the feature vector ***y***_*k*_, which is the LBP histogram. The feature vectors for two windows, *A* and *B*, are then compared using the cosine similarity measure,

$$s\left(y_{A},y_{B}\right)=\frac{y_{A}\cdot y_{B}}{\left|\right|y_{A}\left||\;|\right|y_{B}\left|\right|}.$$

For step 3, we perform a two‐class classification declaring each window of vegetation as being either *grass* or *forb*. Using the cosine similarity measure, the *k*‐th window is compared against templates for each class (*grass* and *forb*) and the class template that best matches (represents) the window is declared as the vegetation type for that pixel; this is a nearest neighbor classifier.

The class templates were obtained by manually annotating a small number of randomly selected images from each site the class. A subset of these windows that contained only *grass* or *forb* was chosen to be the templates. A total of 15 and 10 windows were annotated for Bogong High Plains and Smith Prairie respectively. The low number of annotations makes this approach rapidly deployable to new sites; however, it meant that other classifiers such as support vector machines or random forests could not be utilized.

## 3 DATASETS AND EVALUATION PROTOCOLS

### 3.1 Nutrient network

The automatic estimation of vegetation cover is evaluated on two sites from the Nutrient Network (NutNet) (Borer et al., 2014; Grace et al., 2016; Hautier et al., 2014). Bogong is an alpine grassland situated on the Bogong High Plains (Victoria, Australia) at an elevation of 1,760 m and Smith Prairie is a more productive temperate grassland in Puget Sound (Washington state, United States of America) at an elevation of 62 m.

**Figure 2:**
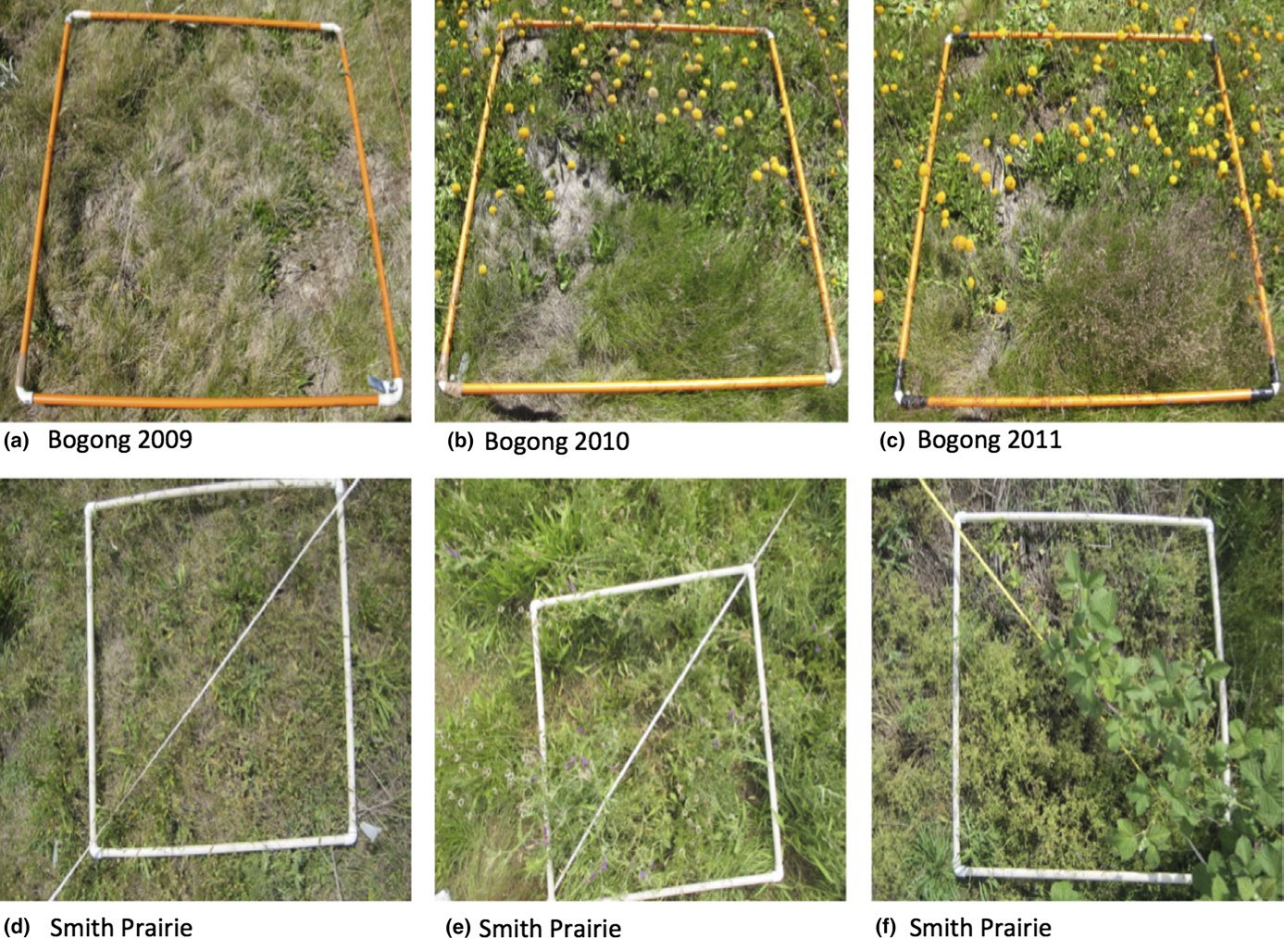
Example quadrat images from the two datasets. (a–c) Show an example of quadrat photographs taken at the Bogong High Plains site over 3 years of the NutNet experiment 2009 to 2011. Photographs (b and c) provide an example of the increase in flowering by forbs in the plots. (d–f) Show an example of quadrat photographs taken at the Smith Prairie site. These three images highlight the different vegetation being estimated. (d) Provides an example of the typical grass present, (e and f) provide an example of the small and large leaf forbs present

Each site had 30 quadrats where photographs were taken and cover was estimated. A PVC frame identified the perimeter of each quadrat. We analyzed photographs of 30 quadrats in each of 3 years (2009–2011) from Bogong and photographs of 29 quadrats for each of 2 years from Smith Prairie (2015–2016)^3^; see Figure 2 for example images. The Smith Prairie images were taken in May with a Canon PowerShot SD1200 IS camera (2015) or Canon PowerShot ELPH 115 IS (2016). The Bogong images were taken with a Canon DIGITAL IXUS 80 IS.

Vegetative cover was surveyed at the Bogong site each year at the start of the growing season in January. Cover was estimated separately for each species, so total cover could sum to >100% because of overlapping canopies. Cover was generally recorded to the nearest percent. At Smith Prairie, the vegetation was assessed in both May (Spring) and June (Summer) to capture the variation associated with species turnover at this higher productivity site characterized by a longer growing season. Where a species was recorded during both census times, the highest cover estimates were retained as the estimate.

As each site was part of the NutNet study, the experimental treatments were the same. There were ten treatment types

$$T_{\text{type}}=\left\{\text{Control},\;\text{Fence},\;K,\;N,\;\text{NK},\;\text{NP},\;\text{NPK},\;\text{NPK}+\text{Fence},\;P,\;\text{PK}\right\}$$

that include the impact of the boundary (Fence) as well as the addition of nitrogen (N), phosphorus (P), potassium (K), and combinations thereof. Treatments were initiated in 2009 at Bogong site and in 2008 at the Smith Prairie site. Fertiliser application will generally increase species cover and generally increase the cover of graminoids particularly in areas that are fenced from vertebrate consumers, but this response can vary depending on the type of grasslands, and annual climatic conditions such as rainfall and temperature.

### 3.2 Performance analysis

To evaluate the effectiveness of the automatic system in comparison with vegetation surveys conducted by field ecologists, we employed two performance measures. First, we evaluate how accurately the automated approach estimates the amount and type of vegetation present compared with visual estimates from ecologists. Second, we evaluate whether inferences were consistent between the two methods, the automated system and ecologists, by comparing analyses of treatment effects using automated and expert (ecologist)‐derived data.

To measure how accurately the automated system estimates the amount and type of vegetation, we use two measures: (1) the correlation between the automated system and the field ecologist across all of the quadrats and (2) calculate Cohen’s kappa, inspired by (McHugh, 2012), to measure the classification agreement for change in coverage (either increasing or decreasing) between the automated system and ground truth (ecologist) across the years. Cohen suggested the following interpretations for kappa values: indicating no agreement ≤0, none to slight agreement 0.01–0.20, fair agreement 0.21–0.40, moderate agreement 0.41–0.60, substantial agreement 0.61–0.80 and almost perfect agreement 0.81–1.0. We treat the coverage estimates from the field ecologist as the true estimate of total vegetation (*V*_veg._), *forb* (*V*_forb_) and *grass* (*V*_grass_). From the automated system, we obtain the percentage of pixels that are vegetation (*A*_veg._) within the quadrat (the region defining the interior of the quadrat is marked manually). Each pixel classified as being vegetation is then declared as being either *grass* of *forb*, this is the percentage of declared vegetation that is either *grass* (*A*_grass_) or *forb* (*A*_forbs_). To convert these percentages into a coverage estimate, we normalize using the percentage of total vegetation,

$$A_{\text{grass}}^{*}=A_{\text{grass}}*A_{\text{veg.}},\;A_{\text{forbs}}^{*}=A_{\text{forbs}}*A_{\text{veg.}}.$$

This allows us to normalize the estimates for *grass* and *forb*; however, it does not allow us to normalize the estimate for the amount of total vegetation.

To measure the consistency of inferences, we use linear mixed effect models (LMEMs) (Bates, Maechler, & Bolker, 2012; Bolker et al., 2008) to measure the impact of treatment types. We used LMEMs to model how the proportion of *forb*,* grass* and vegetation are influenced by the treatment at the plot site. A model, *H*_0_, is derived for each treatment type which accounts for the effect that treatment had upon the amount of coverage, and the random effects associated with the quadrats being nested in the year. For the *j*‐th coverage estimate (*grass*,* forb* or vegetation)

$$H_{0}:{\text{Coverage}}_{j}∼\text{Treatment}+\left(1|\frac{\text{Year}}{\text{Plot}}\right),\forall j$$

$${\text{Coverage}}_{i,j}=\beta_{0}+\sum_{k=1}^{9}\beta_{k}\left(h_{i}=={\text{Treatment}}_{k}\right)+Z_{i}u_{i}+\varepsilon_{i}$$

$$Z_{i}∼N_{q}\left(0,D\right)$$

$$\varepsilon_{i}∼N_{n_{i}}\left(0,\Sigma_{i}\right),$$

where *Z*_*i*_ is the random effect parameter and *D* is the covariance matrix of random effects, *u*_*i*_ is the covariate associated with each treatment for the random effects, β_0_ is the control treatment, ε_i_ is the irreducible error, Σ_*i*_ is the covariance matrix of the irreducible errors, and *h*(.) is a binary indicator function.

## 4 RESULTS

### 4.1 Estimating vegetation and group coverage

**Figure 3:**
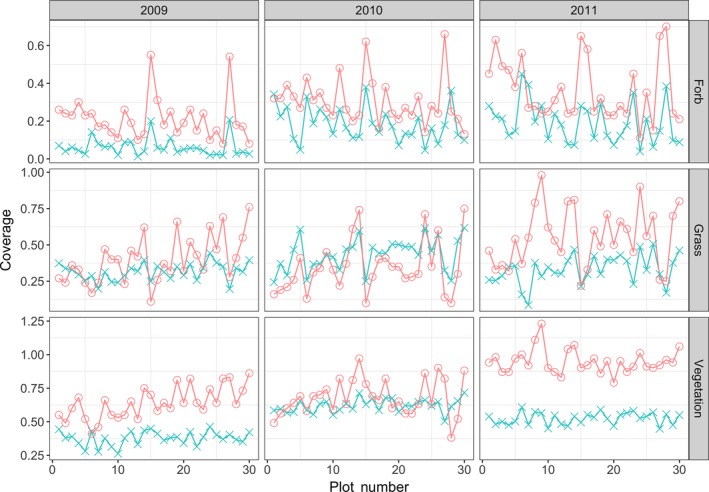
Cover estimates conducted in the field (Ground Truth, salmon) versus the estimates generated by the algorithm (Model, aqua) for the Bogong High Plains dataset. Each column shows results from a different year, from left to right 2009, 2010 and 2011. Each row corresponds to the estimated forb, grass, and total vegetation coverage, moving from top to bottom. Points shown within each panel correspond to one of 30 quadrats that were measured over the 3 years

**Figure 4:**
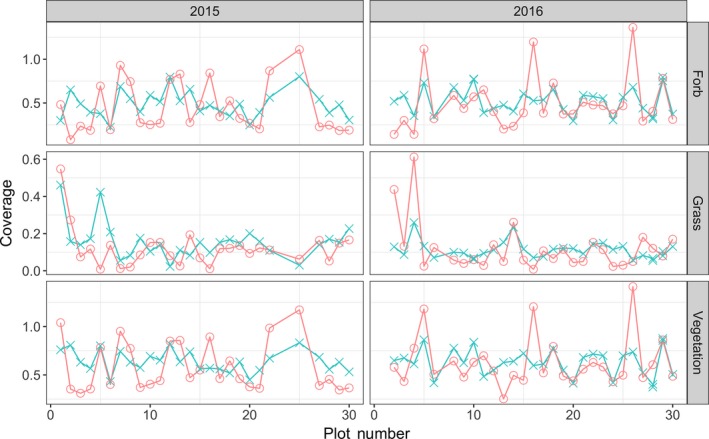
Cover estimates conducted in the field (Ground Truth, magenta) versus the estimates generated by the algorithm (Model, cyan) for the Smith Prairie dataset. Each column presents the results for a particular year, left is 2015 and right is 2016. Each row corresponds to, from top to bottom, the estimated forb, grass and total vegetation coverage. Points shown within each panel correspond to one of 27 and 28 (of 30) quadrats that were measured over 2015 and 2016, respectively

**Figure 5:**
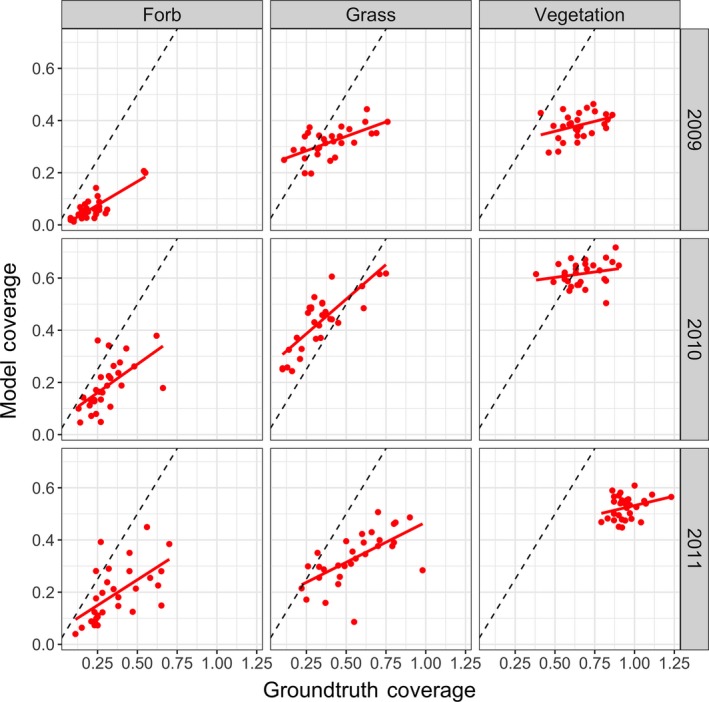
Scatter plot for the Bogong High Plains dataset. Each column corresponds to, from left to right, forb, grass and vegetation. Each row corresponds to a different year, from top to bottom, 2009, 2010, and 2011. The black dashed line represents a Pearson’s correlation of 1 and the solid red line represents the linear fit to the points

**Figure 6:**
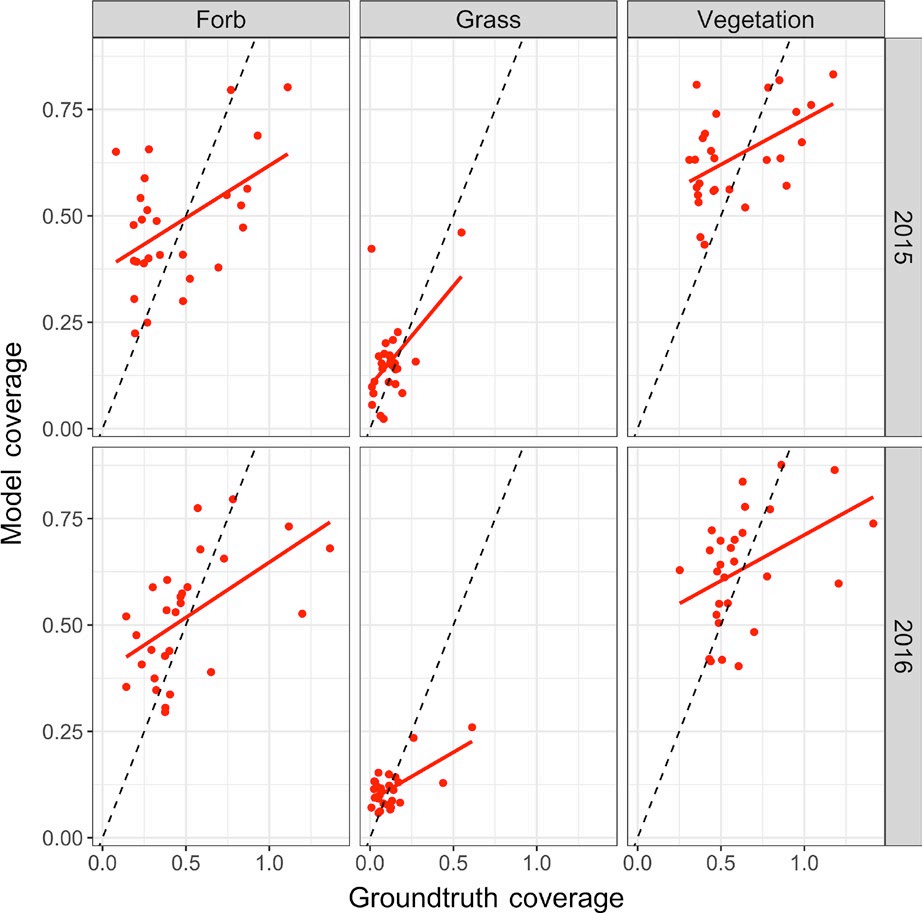
Scatter plot for the Smith Prairie dataset. Each column corresponds to, from left to right, forb, grass and vegetation. Each row corresponds to a different year, from top to bottom, 2015 and 2011. The black dashed line represents a Pearson’s correlation of 1 and the solid red line represents the linear fit to the points

We found reasonable Pearson’s correlations between cover estimates made by the field ecologist(s) and automated system (Table 1). For Bogong, across the 3 years, the correlation for *grass* and *forb* is 0.59 or greater. The correlations for Smith Prairie, for *grass* and *forb*, are slightly lower but always 0.48 or greater. This trend can also be seen in Figure 3, for Bogong, and Figure 4, for Smith Prairie, which present the estimated coverage for the automated system and field ecologist. Equivalent scatter plots are given in Figure 5, for Bogong, and Figure 6, for Smith Prairie.

**Table 1:** Correlations between the automated system and the ground truth from an in‐field ecologist

	Year	Grass	Forb	Vegetation
Bogong High Plains	2009	0.61	0.83	0.41
	2010	0.82	0.60	0.34
	2011	0.62	0.59	0.29
Smith Prairie	2015	0.52	0.48	0.51
	2016	0.63	0.55	0.41

Examining the kappa coefficients (Table 2) for coverage through the years indicates that forbs are the most consistent group coverage between the ground truth and the automated system. For Bogong, across the 3 years, there is fair agreement for forbs, 0.34, but no agreement for vegetation and grass, −0.31 and −0.43, respectively. For Smith Prairie, across the 2 years, there is moderate agreement for grass, 0.43, fair agreement for forbs, 0.31, and slight agreement for vegetation, 0.02.

**Table 2:** Kappa coefficients through the years between the automated system and the ground truth from an in‐field ecologist. The Kappa coefficient measures how successfully the automated system measures the change in cover (increase or decrease) over time

	Bogong High Plains	Smith Prairie
Forbs	0.34	0.31
Grass	−0.43	0.43
Vegetation	−0.31	0.02

The lowest correlations occur for total vegetation coverage for Bogong, with the lowest correlation being 0.29 for 2011. For Bogong, the 2011 growing season experienced a spike in vegetation cover most likely due to increased rainfall, but this peak cover change was not detected by the automated system. To better understand why this might be the case, we examined the images from 2011 of Bogong and note there is a consistent increase in not just vegetation but also of flowers, as can be seen in Figure 2. A similar trend can be seen from 2009 to 2010, where the vegetation increases, but so do the instances of flowers. Unfortunately, the current automated system does not currently classify flowers as vegetation as they are not green. This impacts the total vegetation estimate as well as the estimates for *grass* and *forb*. The images from Smith Prairie also captured plants flowering; however, their flowers were green and so this trend is not present.

A limitation with the automated system is that it is unable to detect some of the peaks for vegetation coverage, in particular when the estimated coverage exceeds 1.0. The automated system is unable to provide a coverage value greater than 1.0 as the vegetation coverage is the percentage of pixels that are considered to be vegetation (green), consequently the maximum value is 1.0. This explains the discrepancies in the Smith Prairie data for 2016, see Figure 4.

### 4.2 Impact of treatment type

**Figure 7:**
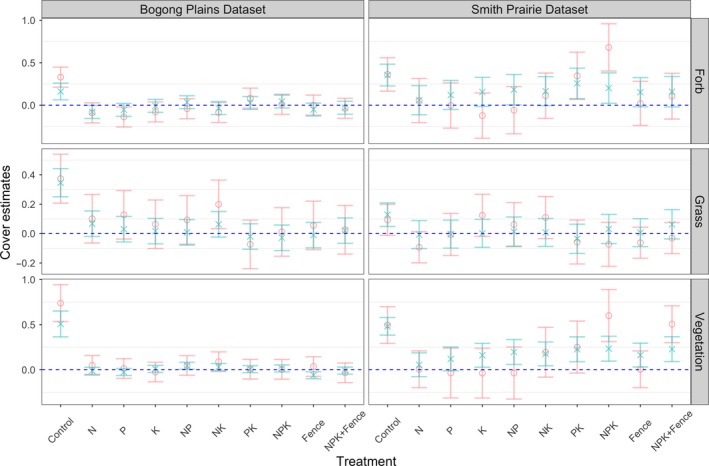
Linear mixed effects model (LMEM) response effect sizes (intercepts) for forb, grass, and vegetation, respectively, for the Bogong High Plains (left) and Smith Prairie (right) datasets. Groundtruth based on the field ecologist estimates (Ground truth, salmon) and the results from the algorithm (model, aqua). Each row corresponds to, from top to bottom, the estimated forb, grass, and total vegetation coverage. Included is the 95% confidence interval for each value

The field ecologist(s) and automated system are reasonably correlated when we examine cover response to the treatments (i.e., nutrient and/or exclusion of vertebrate consumers) using a LMEM (Figure 7). When examining Figure 7, we concluded there was no significant change if the variance of the model, blue line for ecologist and red line for the model, contained zero. We concluded there was a significant change if the 95% confidence interval did not include zero. We then examined the results of Figure 7 to determine when the same conclusion was made by the ecologist and the model.

For the *forb* and *grass* groups, it can be seen that the same conclusion is made for eight of the nine treatments (excluding control) for both Bogong High Plains and Smith Prairie. A similar trend can be seen for Bogong High Plains for vegetation coverage with the same conclusion for seven of the nine treatments. However, for Smith Prairie, the vegetation coverage leads to a different trend with the same conclusion for just four of the nine treatments.

We attribute the poor performance for vegetation coverage for Smith Prairie to the fact that the automated system describes vegetation coverage as the percentage of pixels that contain vegetation. This is not equivalent to what a field ecologist is estimating. In particular, this analysis focused on dominant species at the time the images were taken, whereas the ecologist estimated cover in both spring and summer, and some species were subordinates that grew beneath the dominant species and thus were not evident in the images.

## 5 DISCUSSION

In this study, we have presented an automated approach that can estimate vegetation cover from digital images of quadrat taken with a regular camera and can classify that cover into two functional groups: *grass* and *forb*. The automated system‐ and field‐based measurements from ecologists were reasonably well correlated even though the grasslands were ecologically very different—one a relatively low stature subalpine grassland in Australia and the other a high stature and more productive lowland grassland in the USA. We found that using the automated system would lead to similar treatment conclusions. Based on *grass* and *forb* coverage, nine of 10 of the interpretations of treatment responses would be the same at each of the two sites whether using the automated photograph interpretation process or the field ecologist data, although inference was less consistent for total vegetation cover. This demonstrates the potential of an automated system to facilitate the interpretation of quadrat imagery and provide important information with similar consistency and accuracy over space and time to a field ecologist.

A particularly exciting aspect of this work for ecology is the prospect to increase the spatial and temporal resolution of vegetation studies. An automated system like this could be applied to images of larger areas than quadrats. For example, images could be obtained from a UAV during a short time window, thus avoiding errors in interpretation that arise from species turnover and the phenological changes that occur during a long field season of vegetation sampling. In fact, those phenological changes could also be studied at large scales by taking additional images throughout the season.

There are three limitations with the current automated system. First, the photographs convert a three‐dimensional system into a two‐dimensional image, which limits the ability to assess understory cover. Also, we have assumed that the scale of the images is similar for a given site and that the viewing angle of the camera does not impact the overall area that a pixel is responsible for. Future work could alleviate these assumptions by normalizing the images. This image normalization could include normalizing the image to a constant size based on the estimated quadrat location, accounting for lens distortion and exploiting the known size of the quadrat. Second, having lost the three‐dimensional information, total vegetation cover is given as a percentage of pixels that are considered occupied by vegetation. However, when an ecologist performs a survey, a pixel region could contain both *grasses* (or other *graminoids*) and *forbs*, and this can lead to coverage estimates greater than 1.0, which is not possible with the automated process developed here. This means that simply declaring that a pixel contains vegetation is insufficient to accurately estimate the amount of vegetation present within a quadrat. Third, the system can be confused by the presence of flowers leading it to conclude that less vegetation is present than is actually the case. Regardless of these limitations, we have shown an ecologist’s assessment of responses to treatments over space and time would be remarkably similar to the proposed automated method.

Future work could address this by performing multilabel classification or regression for each pixel, such that a single pixel in the image could represent both *grass* and *forb*. Such an approach would better match the process undertaken by field ecologists and would potentially overcome the limitation that the current automated system can only provide a percentage of total vegetation (it is unable to produce a coverage estimate greater than 1.0).

Finally, our approach does not yet have the sensitivity to reliably estimate cover for all species individually or to measure finer detail functional groups such as the difference between forbs and legumes and grasses and sedges.

Nevertheless, this method illustrates how cover of different vegetation types can be easily collected and then quantitatively evaluated to record changes over time. In particular, these methods offer a reliable alternative or complimentary approach to estimating cover that is more repeatable and likely to be more reliable over the long term and so is especially useful for long‐term monitoring We recommend that top–down photographs be taken as a standard adjunct to any quadrat‐based measurement, and possibly even top–down video of an entire site using transects. While aggregated cover estimates can reliably be made, photographs also provide a permanent picture of vegetation at a point in time that can be analyzed retrospectively. As image analysis methods develop, the photographs can be reanalyzed to reflect these advances and so enable improved measurement of historical data.

## CONFLICT OF INTEREST

None declared.

## AUTHORS CONTRIBUTION

The following is a summary of the author contributions. The first author, Dr Chris McCool, designed, implemented, and ran the computer vision algorithms. He wrote much of the manuscript and collated the input from the other authors. The second author, Mr James Beattie, ran the statistical interpretation of the results comparing the computer vision algorithms and the ground truth (provided by in‐field ecologists). He also wrote considerable content for the manuscript. The third author, Prof. Michael Milford, provided insight into the development of the computer vision algorithms and provided content for the computer vision part of the manuscript as well as feedback during the manuscript’s development. The fourth author, Assoc. Prof. Jonathan Bakker, provided invaluable feedback on the experimental results, made available the Smith Priairie dataset and provided feedback throughout the manuscript’s development. The fifth author, Dr Joslin Moore, provided invaluable feedback on the experiments results, made available the Bogong Highplains dataset and provided feedback throughout the manuscript’s development. The sixth author, Assoc. Prof. Jennifer Firn, inspired the initial work and provided constant feedback and support. She contributed to writing major sections of the manuscript, in particular the ecological interpretation of the results.

## ENDNOTES

1 The code and data used is available through the following link: https://tinyurl.com/automation-ecv.

2 Initial experiments found no improvement in performance when using the full covariance matrix.

3 30 quadrats were examined by a field ecologist from Smith Prairie; however, only 27 and 28 images were used, from 2015 to 2016, respectively, as the other images had inconsistent top–down views of the quadrats.
